# Signaling Molecules Regulating Pancreatic Endocrine Development from Pluripotent Stem Cell Differentiation

**DOI:** 10.3390/ijms21165867

**Published:** 2020-08-15

**Authors:** Hui Huang, Taylor N. Bader, Sha Jin

**Affiliations:** 1 Department of Biomedical Engineering, Thomas J. Watson School of Engineering and Applied Sciences, State University of New York at Binghamton, Binghamton, NY 13902, USA; hhuang76@binghamton.edu (H.H.); tbader1@binghamton.edu (T.N.B.); 2 Center of Biomanufacturing for Regenerative Medicine, State University of New York at Binghamton, Binghamton, NY 13902, USA

**Keywords:** signaling molecules, islet cells, extracellular matrix, human pluripotent stem cells, pathways, maturation

## Abstract

Diabetes is one of the leading causes of death globally. Currently, the donor pancreas is the only source of human islets, placing extreme constraints on supply. Hence, it is imperative to develop renewable islets for diabetes research and treatment. To date, extensive efforts have been made to derive insulin-secreting cells from human pluripotent stem cells with substantial success. However, the in vitro generation of functional islet organoids remains a challenge due in part to our poor understanding of the signaling molecules indispensable for controlling differentiation pathways towards the self-assembly of functional islets from stem cells. Since this process relies on a variety of signaling molecules to guide the differentiation pathways, as well as the culture microenvironments that mimic in vivo physiological conditions, this review highlights extracellular matrix proteins, growth factors, signaling molecules, and microenvironments facilitating the generation of biologically functional pancreatic endocrine cells from human pluripotent stem cells. Signaling pathways involved in stepwise differentiation that guide the progression of stem cells into the endocrine lineage are also discussed. The development of protocols enabling the generation of islet organoids with hormone release capacities equivalent to native adult islets for clinical applications, disease modeling, and diabetes research are anticipated.

## 1. Introduction

Diabetes has become one of the most common diseases around the world. In diabetic patients, glucose homeostasis cannot be obtained due to the dysfunction of pancreatic islets. Currently, the donor pancreas is the only source of human islets, restricting the availability of islet supply [1]. Although insulin therapy is a common treatment for diabetes, it is not a cure. Therefore, it is vital to develop renewable sources of islets for diabetes research and treatment. The pluripotency and infinite self-renewal features of human stem cells offer an unlimited source for generating islet tissue. In the past two decades, extensive efforts have been made to derive insulin-secreting cells and islet-like organoids from human embryonic stem cells (hESCs) and/or human induced pluripotent stem cells (iPSCs) in vitro [2,3,4,5,6]. Recently, the generation of islet organoids consisting of multiple hormone-secreting islet cell types from human pluripotent stem cell (hPSC) differentiation, including both iPSCs and hESCs, has been reported [7,8].

Human pancreatic islets are mainly composed of four types of cells, which are glucagon-secreting cells (α-cells), insulin-secreting cells (β-cells), somatostatin-secreting cells (δ-cells), and pancreatic polypeptide-secreting cells (PP-cells) [9]. The dysfunction of any of these cells will cause dysglycemia. β-cell destruction by the immune system results in type I diabetes (T1D). The overexpression of glucagon due to the dysfunction of α-cells is frequently found in T1D patients [10]. Though PP has no effect on insulin secretion, it has been shown to have an effect on inhibiting glucagon secretion at low glucose concentrations [11]. A mouse model study showed that in somatostatin (SST) knock-out mice, the inhibition of glucagon secretion by glucose level change was not obvious [12]. Hence, SST and PP primarily regulate blood glucose homeostasis by affecting glucagon secretion.

On the other hand, the in vitro generation of endocrine cells from hPSC differentiation is based on stepwise protocols to mimic the natural developmental progression. Stem cells are induced to differentiate into definitive endoderm, posterior foregut, pancreatic progenitors, endocrine progenitors, and, finally, islet cells. This differentiation process relies on a variety of signaling molecules to guide the differentiation pathways, as well as culture microenvironments to mimic in vivo physiological conditions. This review highlights signaling molecules, including the extracellular matrix proteins, growth factors, and small molecules, that regulate cell signaling pathways for the generation of physiologically functional islet cells from hPSC differentiation. It also discusses the effects of culture microenvironments on the generation of mature islet cells from stem cells.

## 2. Molecules Promoting the Generation of Functional β-Cells from Human Pluripotent Stem Cells

To date, substantial studies have been focused on differentiating insulin-secreting β-cells for the realization of stem cell-derived β-cell transplantation to cure diabetes. Several molecules and signaling pathways have been identified to enhance in vitro hPSC differentiation into glucose-responsive insulin-secreting cells. For example, enhancing Wingless and Int-1 (WNT) [13], nodal growth differentiation factor (NODAL) [14], and transforming growth factor β (TGF-β) signaling [13] during the generation of definitive endoderm could increase the yield of this lineage. Inhibiting the activin receptor-like kinase 5 (ALK5) [15], bone morphogenetic protein (BMP) [4], and Sonic hedgehog (SHH) [16] signaling, and augmenting retinoic acid (RA) signaling could lead to the formation of pancreas endoderm. Continual inhibition of ALK5 and SHH signaling and inducing BMP signaling can induce the formation of pancreatic endocrine cells. Therefore, the timing to stimulate or suppress a signal is critical during the stepwise differentiation period, which commonly takes between 24 and 35 days.

From a mechanistic point of view, the transcription factors Pdx1, Nkx6.1, MafA, and NeuroD regulate the expression of the insulin gene. Interestingly, the expression of Pdx1 is universal in the early stage of pancreatic development and, in both endocrine and exocrine cells, MafA is specifically expressed in β-cells [17,18]. MafA-deficient mice showed symptoms of diabetes mellitus [19] and islet cells of type II diabetes (T2D) patients expressed MafA at a low level [20]. Hence, MafA is a crucial factor in β-cell formation and maturation. MafA has been found to specifically bind to RIPE3b to regulate insulin gene expression according to glucose concentration [21,22]. Therefore, elevating the expression of MafA in insulin-producing cells may enhance the cell’s maturity. Aguayo-Mazzucato and his coworkers have examined this hypothesis. They used triiodothyronine (T3), a type of thyroid hormone, to treat pancreatic endoderm cells derived from hESCs. After differentiation toward endocrine cells, the expression levels of MafA and insulin secretion at a high glucose concentration (16.8 mM) were increased five-fold compared to those of untreated cells [23]. In addition, the ALK5 inhibitor is able to enhance MafA expression as well [20]. The addition of T3 and ALK5 inhibitor together to differentiation medium from stage 5 (pancreatic endoderm precursor cells) to stage 7 (insulin-expressing cells) resulted in higher expression levels of MafA [2].

In addition to MafA, Nkx6.1 is one of the few transcription factors that has been detected in the adult pancreas [24]. Nkx6.1 is directly involved in the regulation of the glucose metabolic gene Glut2 [25]. The suppression of Nkx6.1 in β-cells resulted in the impairment of β-cells’ glucose sensitivity and intolerance [26]. The expression of Nkx6.1 is also important for inducing the endocrine precursors’ progression into β-cells rather than other islet cell types. It acts as a repressor of the expression of Aristaless-related homeobox (Arx) which is a transcription factor of α-cells [24]. Memon and his coworkers reported that treatment with RA and fibroblast growth factor 10 (FGF10) can augment the expression of Nkx6.1 [27]. The FGF10 signal was applied from the formation of pancreatic foregut through to the end of the formation of pancreatic endoderm during stepwise differentiation, and the RA signal was exerted during the formation of pancreatic endoderm. The enhanced expression of a PDX1^–^/NKX6.1^+^ population can be achieved by manipulating the re-plating density of endodermal cells, and further differentiation into endocrine progenitors [27]. Recently, it was discovered that blending type V collagen with Matrigel as coating substrates for iPSC endocrine differentiation can significantly augment the gene expressions of Pdx1 and Nkx6.1, leading to glucose-responsive insulin and glucagon secretion in iPSC-derived islet organoids [8]. In another study, it was found that the spontaneous clustering of cells, measuring less than 500 µm in diameter, can significantly increase the expression of NKX6.1 and PDX1 compared to cells cultured in monolayers, although the mechanisms underlying the effect of aggregate size on these key marker expressions is unknown [28].

As mentioned above, the expression of Pdx1 is another important signal for the formation of the pancreas [29]. Several signaling pathways and molecules have been examined for the induction of Pdx1 expression, such as BMP signaling, the activin family, the MAF bZIP transcription factor (MAF) family, and RA [30,31]. activin A and RA are commonly used to promote the formation of pancreatic precursor cells in many protocols for generating β-cells from hPSCs [32]. The experiments on hESCs showed that, without the addition of RA, FGF could not induce the formation of Pdx1^+^ cells. Adding RA sooner after the formation of definitive endoderm and keeping it for the whole differentiation process could achieve the highest expression of Pdx1 [33]. Differentiating iPSCs in an activin-containing gelatin-poly(lactide-co-glycolide) scaffold followed by the addition of RA allowed for the generation of glucose-responsive insulin secretion cells [34]. Epidermal growth factor (EGF) is another protein that has been recognized as a signaling molecule for directing hPSC differentiation into insulin-secreting cells [4]. It plays a role in guiding the successful formation of pancreatic progenitors, resulting in significant enhancement of the expression of Pdx1 [29].

Exendin-4 is an analog of glucagon-like peptide-1 (GLP-1) [35]. Studies on rat islet β-cells have shown that exendin-4 increases the biosynthesis of glucose-stimulated proinsulin and stimulates the process of insulin-mediated glucose uptake [36,37]. Therefore, exendin-4 has been used to promote β-cell maturation in the late stages of differentiation. The Movassat group found that treating human pancreatic islet-like cell clusters with exendin-4 for 4 days can upregulate the expression of PDX1 protein [38]. After the transplantation of the islet-like cell clusters into mice together with exendin-4 treatment, these cell clusters displayed glucose-dependent characteristics [38]. In another study, after treating hESC-derived endocrine progenitors with exendin-4, along with other factors, for over 10 days [39], there were 35% mono-hormonal insulin^+^ cells and these cells were sensitive to glucose level, as assessed by a glucose-stimulated insulin secretion (GSIS) assay. Under high glucose (16.5 mM) stimulation, the insulin secretion level was about three times higher compared to the low glucose (2.8 mM) stimulation [39]. Table 1 summarizes the molecules that have been widely applied for promoting insulin-secreting β-cell development from stem cells.

**Figure 1:**
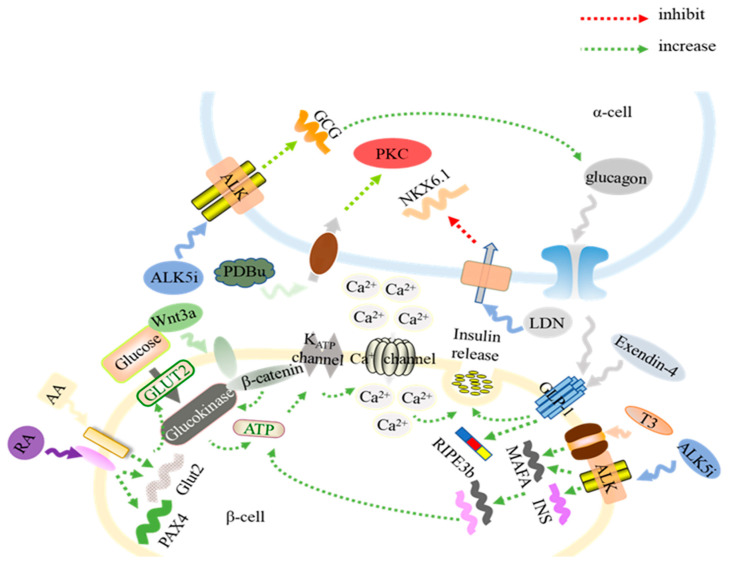
The mechanisms of signaling molecules that enhance the generation of glucose-responsive insulin (INS)-secreting β-cells and functional α-cells. Treatment with retinoic acid (RA) and activin A (AA) increases the expression of Glut2, which transports glucose into β-cells. After a series of reactions, there is Ca^2+^ flux into β-cells, inducing insulin secretion. Wnt3a enhances the process of glucose intake and insulin secretion. Treatment with activin receptor-like kinase 5 (ALK5) inhibitor (ALK5i) and triiodothyronine (T3) enhances the expression of MafA. The ALK5i also promotes the transcription of glucagon (GCG) in α-cells. The paracrine of glucagon and exendin-4 stimulates the formation of RIPE3b, which positively impacts on insulin secretion. Treating pre-α cells with LDN193189 (LDN) decreases the expression of NKX6.1, resulting in an induction of differentiation into mono-hormonal α-cells. The protein kinase C (PKC) activator, phorbol 12,13-dibutyrate (PDBu), also augments the generation of pre-α cells into α-cells.

The aforementioned signaling molecules were speculated to be involved in promoting the development of mature insulin-secreting β-cells from hPSCs, as illustrated in Figure 1. In the β-cells, GLUT proteins are sensitive to the glucose levels and are in charge of the transportation of glucose into β-cells. Once glucose is transported to the cells, glucokinase facilitates the phosphorylation of glucose and increases the ATP/ADP ratio [42], leading to the closure of the K_ATP_ channel and, consequently, increasing membrane resistance and opening the calcium ion (Ca^2+^) channel [43]. The increased Ca^2+^ concentration triggers the exocytosis of insulin. Ca^2+^ promotes the fusion of the membrane of insulin granules with the plasma membrane [44,45]. Therefore, the Ca^2+^ influx of insulin-secreting β-cells in response to glucose level change is one of the characteristics of β-cells (Figure 1). The glucose-induced Ca^2+^ influx oscillation of hPSC-derived β-cells has been measured and quantitatively assessed through Ca^2+^ imaging and data analysis for the in vitro characterization of stem cell-derived β-cell physiological function and the degree of maturation [3,46].

## 3. Signaling Pathways Involved in In Vitro Islet β-Cell Development

Wnt signaling pathways play a crucial role in regulating the maturation of in vitro-derived β- cells. There are three kinds of Wnt signaling pathways: the canonical Wnt pathway, the noncanonical planar cell polarity pathway, and the noncanonical Wnt/calcium pathway. Non-canonical Wnt/planar cell polarity (PCP) signaling has a positive effect on in vitro β-cell maturation [47]. Nevertheless, when iPSC-derived endocrine cells were stimulated by either canonical or non-canonical Wnt signaling molecules, such as WNT3A, WNT4, WNT5A, and WNT5B, the Wnt signaling failed to improve the maturity of iPSC-derived endocrine cells [41]. Interestingly, when the cells’ endogenous Wnt signaling was inhibited by tankyrase inhibitor G007-LK (TKi), the fraction of mono-hormonal cells increased (Table 1) [41]. Global proteomics of TKi-treated iPSC-derived endocrine showed a proteomic signature more similar to that of adult human islets. This study suggested that the inhibition of endogenous Wnt may help β-cell maturation. However, it is unclear whether the iPSC-derived endocrine cells after the inhibition of Wnt signaling treatment possess glucose responsiveness for insulin secretion [41].

Keratinocyte growth factor (KGF) is a member of the fibroblast growth factor family that can stimulate ductal cell proliferation [48,49]. KGF activates the protein kinase B (PKB), also known as Akt, signaling pathway and can increase the β-cell population [50]. KGF was one of the signaling molecules widely used in stepwise differentiation media to permit the development of pancreatic progenitors from hPSCs, as signposted by the high expression of PDX1 and NKX6.1 [40,51,52]. The dual positive pancreatic progenitors accounted for up to 67% of the total cell population [40]. Similar results were also reported by other groups [3]. The successful generation of pancreatic progenitors is a prerequisite for mature β-cell development from hPSCs [3].

To investigate the role of RA signaling in β-cell development, it was found that RA synthesizes the enzyme retinaldehyde dehydrogenase (Raldh1), which is expressed during pancreatic development when β-cells are generated [53]. There was a three-fold increase in the relative number of insulin^+^ cells in cultures containing 25 nM of RA during endocrine cell differentiation. The findings implied that RA promotes the generation of insulin^+^ cells [53]. In addition, the effect of RA on β-cell development was investigated by examining its influence on the pro-endocrine gene Ngn3. There was a four-fold increase in numbers of Ngn3^+^ cells in the presence of RA. Furthermore, there was a three-fold increase in NeuroD expression under RA signaling after the formation of Ngn3^+^ cells. Hence, RA triggers the expression of Ngn3, followed by the expression of NeuroD [53].

## 4. Microenvironments That Facilitate the Formation of β-Cells from Stem Cell Differentiation

A line of studies demonstrated that two-dimensional (2D) monolayer cultures do not provide a microenvironment for the generation of functional, mature endocrine cells. Cells cultured in 2D have low cell–matrix and cell–cell interactions and these cells lack spherical morphology [7,54]. In contrast, a three-dimensional (3D) microenvironment could augment hPSC progression into endocrine lineages [6,7]. A mixture of polycaprolactone and polyvinyl alcohol (PCL/PVA) has been employed to construct biodegradable nanofiber scaffolds to differentiate hPSCs into pancreatic β-cells. PCL was selected for scaffolding because of its biocompatibility, biodegradability, hydrophobicity, and high mechanical stability. However, PCL has poor cell affinity. Blending it with PVA improves this, as PVA is hydrophilic and biodegradable. Cells produced in this platform expressed glucagon, insulin, PDX1, and NGN3 at significantly higher levels than those expressed in 2D cultures [54]. It is anticipated that biodegradable and biocompatible scaffolds may imitate in vivo environments, as well as support extracellular matrix (ECM)–cell and cell–cell interactions, which are vital for the generation of mature, functional pancreatic β-cells [6].

A hydrogel, designated as Amikagel, has been reported to be used for a controlled and spontaneous aggregation of hESC-derived pancreatic progenitor cells into homogenous spheroids [55]. The formation and aggregation of these spheroids increased the expression of PDX1 and NKX6.1, as well as the percentage of cells that co-expressed these markers. In addition, the Amikagel facilitated the aggregation of these cells with supporting endothelial cells, yielding self-organized multicellular pancreatic organoids, which were closer to native islet physiology in terms of heterogeneity. The hydrogel platform induced the spontaneous differentiation of the pancreatic progenitor spheroids into β-like cells, displaying an expression of *C*-peptide protein and the ability of in vitro glucose-stimulated insulin production.

In addition, a new engineering platform currently being deployed to generate islet organoids from iPSCs is combining the stem cell differentiation principles with an organ-on-a-chip platform. An organ-on-a-chip microfluidic device has been developed to facilitate the formation of functional embryonic bodies from iPSCs and 3D islet organoids [56]. These organoids exhibited increased expression of PDX1 and NKX6.1 at both gene and protein levels, as well as increased insulin secretion level and Ca^2+^ flux in response to glucose stimulation. This study suggested that the biomimicry of mechanical cues in culture is of importance for improving islet organoid function and maturation [56].

## 5. Molecules Critical for the Generation of Functional α-Cells from hPSC Differentiation

In human islets, the population of α-cells ranges from 10 to 65% [57]. They secrete glucagon and GLP-1 [58]. The paracrine of glucagon has been proven to regulate insulin secretion [59]. Glucagon can activate the GLP-1 receptor on the β-cells and enhance the secretion of insulin [60] (Figure 1). In T1D patients, not only are their β-cells destroyed by immune cell attack, but also their ability to secrete glucagon properly is disabled by the dysfunction of α-cells [61]. Hence, the generation of α-cells from hESCs has been studied as well. Current hPSC differentiation protocols permitted the generation of both insulin-secreting β-cells and glucagon-secreting α-cells in a pancreatic endocrine lineage by using molecules to control signaling pathways, as shown in Figure 1 [2,3,7,8,62]. For instance, applying LDN193189 (LDN), an inhibitor of BMP signaling, to the middle stage of the differentiation process could suppress the expression of NKX6.1 and induce the development of α-cells [63] (Figure 1, Table 2). As a result, cells were secreting both insulin and glucagon at the end of hESC differentiation [7,8]. In addition, phorbol 12,13-dibutyrate (PDBu) is an activator of protein kinase C (PKC). The addition of PDBu at the later stage of hPSC differentiation could significantly increase the percentage of mono-hormonal glucagon-secreting α-cells [63] (Figure 1, Table 2). This result proved that the activation of PKC is important for the differentiation of functional α-cells. Furthermore, ALK5 inhibitor (ALK5i) has been widely used during the formation of pancreatic endocrine precursors, which allows for increasing the expression of insulin, glucagon, and somatostatin [2,7,8]. The differentiation of hPSCs in the presence of ALK5i allows for upregulating the expression levels of endocrine signature genes (Figure 1) [2]. Therefore, insulin and glucagon are co-expressed in the hPSC-derived pancreatic endocrine.

**Figure 2:**
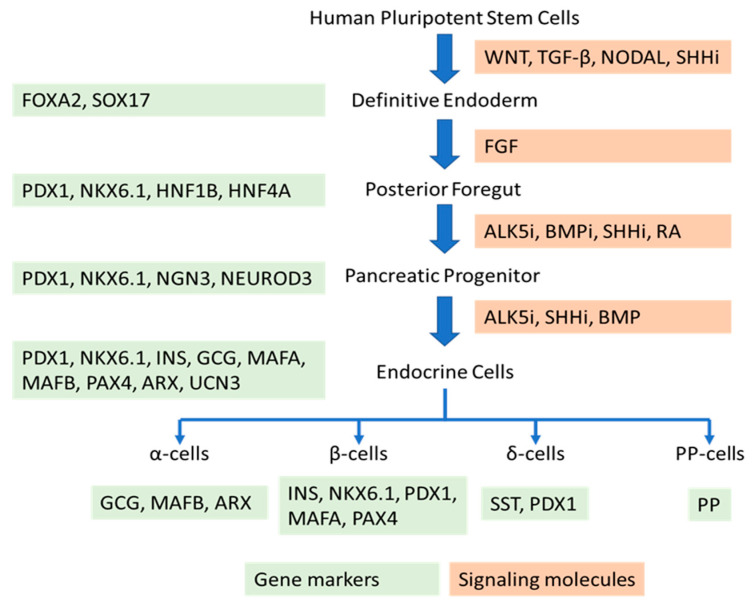
Key signaling molecules (in orange) and marker genes (in green) during the progression of human pluripotent stem cells (hPSCs) to pancreatic endocrine. BMPi: Bone morphogenetic protein inhibitor, ALK5i: ALK5 inhibitor, SHHi: Sonic hedgehog inhibitor.

In another study, it has been investigated whether Neurog3^+^ cells can develop into pancreatic α-cells [64]. Experimental results using Neurog3^+^ knock-in mice unveiled that those Neurog3^+^/Myt1^-^ cells were biased toward an α-cell fate, while the Neurog3^+^/Myt1^+^ cells were biased toward a β-cell fate [64]. However, further study on the identification of whether Myt1 is essential for α- or β-cell development suggested that Myt1 is not a determinant for islet cell type specification, as this gene only marginally affects α- or β-cell differentiation [64]. On the other hand, it has been reported that DNA methyltransferase 1 (Dnmt1) and Arx maintain islet α-cell identity [65]. The loss of Dnmt1 and Arx leads to α-cells’ conversion into β-cells in mice [65]. Inhibiting Dnmt1 in pancreatic progenitors promoted the specification of α-cells [64]. Therefore, a Dnmt1 inhibitor might be applied to the stepwise differentiation protocol to suppress β-cell development and facilitate the formation of α-cells during a later stage of differentiation. The effects of Arx and Pax4 on determining the α- and β-cell fates have been investigated as well [66]. The overexpression of Arx, while inhibiting Pax4, could induce the endocrine progenitor cells to further differentiate into α-cells [67] (Figure 2).

The Rezania group developed a six-stage protocol to differentiate hESCs into pancreas endocrine cells [68]. SHH and BMP signaling were inhibited at an early stage of differentiation to facilitate hESCs to form into pancreatic endocrine cells. After the formation of the foregut progenitor, they allowed the activation of BMP signaling by not adding Noggin to further differentiate the cells to α-cells (Table 2). While the derived mature endocrine cells contained insulin-, glucagon-, somatostatin-, and ghrelin-positive cells, the glucagon protein content was 10-fold higher than that of human islets, while insulin protein content was 10-fold lower than that of human islets [68]. The basal glucagon secretion level was comparable to adult islets. Further culturing decreased the proportion of insulin^+^ and insulin^+^/glucagon^+^ cells, suggesting an effective approach to generate glucagon-secreting α-cells from hPSCs [68]. Table 2 lists the major molecules intensifying islet α-cell development from stem cells.

## 6. Generation of Other Types of Islet Cells

Though α-cells and β-cells comprise a large portion of pancreatic islets, there are other endocrine cells named δ-cells and pancreatic polypeptide cells (PP cells). The cell–cell interactions between α-, δ-, and PP-cells are non-negligible, as such interaction permits islet function and characteristics. δ-cells have dendrite-like extensions similar to neuron cells, causing the formation of a network for cell–cell crosstalk to regulate hormone release [69,70]. In diabetic mice, δ-cells showed a tendency to migrate from the peripheral area to central isles [71]. Since SST usually functions as an inhibitor in islets [12], any changes in the islet architecture will impact the intra-islet paracrine communication. Animal studies showed that an increase in somatostatin causes the reduction of counter-regulatory glucagon secretion for insulin-induced hypoglycemia situations [72,73]. Somatostatin also inhibits the secretion of insulin and glucagon at high glucose concentrations [74,75]. δ-cells have receptors in the SHH pathway called Ptch1. Ptch1 receptors are only found in δ-cells [76]. The proper function of the SHH pathway is important for the development of the pancreas, as mentioned above. Its dysfunction could be one of the causes of T2D. Therefore, developing δ-cells in vitro is also important.

As mentioned above, PDX1 is an important protein that regulates the formation of the pancreas. Its ablation will lead to pancreatic agenesis [77]. PDX1 was recognized as a transcription factor for not only β-cells but also δ-cells [78] (Figure 2). A cell line derived from human fetal islets, designated as TRM-6, was genetically modified to express PDX1 constitutively [79]. As a result, the TRM-6 cells exhibited up to a 100-fold increase in somatostatin gene expression, similar to the expression level of somatostatin in human islets [79]. The cell aggregation culture further permitted the production of somatostatin protein. The study indicated that PDX1 and cell–cell contact synergistically promote islet δ-cell development. Figure 2 highlights the key signaling molecules and marker genes during the progression of hPSCs into pancreatic endocrine tissue.

Pancreatic polypeptide cells, also known as PP-cells, only count for a small part of pancreatic cells. PP-cells are rich in the islets located in the head of the pancreas, while they are scarce in the islets located in the tail of the pancreas [80]. Pancreatic polypeptide is secreted by PP-cells under the control of glucose level, neuropeptides, food intake, and gastrointestinal substances [81]. Food intake or higher glucose levels lead to an increase in pancreatic polypeptide levels [11]. There are some clinical studies which confirm that pancreatic polypeptide plays an important role in regulating glucose in patients with pancreatogenic diabetes [82]. Its function as a treatment for T1D and T2D has been proven in mouse models [83]. Obese mice were injected with bovine pancreatic polypeptide (bPP) in a dosage of 200 µg/day/kg of body weight for 5 days. The injected bPP increased the sensitivity of the animals to exogenous insulin [83].

A molecule of interest for the generation of PP-cells is human activin A. To investigate its effect on the formation of PP-cells, a rat pancreatic exocrine cell line, AR42J, was cultured in the presence or absence of activin A [84]. The gene expression of the pancreatic polypeptide was detectable when cells were cultured in activin A-containing medium, while neither insulin nor glucagon mRNA expression in the cells were detectable. There was a non-detectable level of pancreatic polypeptide expression in the native AR42J cells. In addition, PP^+^ cells treated with activin A maintained their round morphology, as well as showed increased cell survival for three days after the initial treatment of activin A. These results suggested that activin A induces the differentiation of PP^+^ cells from rat pancreatic cells [84].

## 7. Approaches for the Generation of Islet Organoids from hPSCs

### 7.1. Suspension Cultures Enhance the Differentiation of Islet Organoids

While previous studies have primarily focused on generating β-cells from stem cells or progenitors [2,3,85,86], paracrine regulation by the various islet hormones within intact islets is essential for maintaining physiological blood glucose regulation, which is principally mediated through the action of insulin on storage, glucagon on release, and somatostatin on insulin and glucagon secretion [7]. In T1D patients, the damage of β-cells causes the dysfunction of α-cells, which leads to hypoglycemia. Hence, both functional α- and β-cells are required for the cellular therapy of T1D [87]. The interactions between β-cells through gap junctions allow for the synchronization of the glucose response among cells [88,89]. Accordingly, the composition and relative proportion of islet cells have profound effects on regulating pancreatic endocrine cell maturation and their physiological functions in vivo. Therefore, it is highly desired to generate intact islets or islet organoids consisting of all islet cell types from hPSCs for diabetes research and treatment.

Human islets secrete basal insulin when blood glucose levels are less than 3 mM and release increased levels of insulin when blood glucose levels are higher than 11 mM [89]. Interestingly, such glucose-responsive insulin secretion characteristics are only displayed by intact islets. After dispersing the islet cells into individual cells, this feature is impaired. The islet cells that are cultured in monolayer lose their ability to produce hormones [79]. Likewise, studies demonstrated that cell aggregation cultures could enhance the differentiation of hPSCs into pancreatic lineages with enhanced physiological function [51,79]. Accordingly, one of the approaches to produce islet-like organoids is to generate endocrine cells first by a 2D culture platform, followed by the aggregation of cells into clusters and culturing them for an extended time to allow for islet cell maturation. The Kim group induced the differentiation of pancreatic endocrine cells from hESCs by inhibiting TGF-β/Nodal signaling using dorsomorphin and SB431542 in 2D culture [90]. The derived endocrine cells were dissociated and re-seeded to allow the formation of cell clusters. The expression of glucose sensor genes, SLC2A1 and GCK, were increased in clustered endocrine cells. These hESC-derived clustered endocrine cells secreted insulin, responding to glucose concentration change, suggesting the improvement of β-cell maturity by clustering culture. Immunostaining results exhibited that the cell clusters were able to produce insulin, somatostatin, and pancreatic peptide proteins, though glucagon was not detected. The gene expression level of MafB, a transcription factor crucial for glucagon-secreting α-cells, was significantly reduced after the clustering culture of the endocrine cells [90]. While the transplantation of these cell clusters balanced the glucose level in a mouse model, normal sugar levels in the blood could only be leveraged for less than half a month [90].

Another approach to generate islet-like organoids is to develop a microwell culture system to control aggregate size during iPSC islet differentiation [91]. The iPSCs were seeded in the microwell device, which contains hundreds of microwells to allow cells to form aggregates before initiating differentiation. After 27 days of culturing in stepwise differentiation media, immunostaining results revealed that these cells were able to express insulin, glucagon, and somatostatin proteins, although pancreatic polypeptide was not detected [91]. By using this system, the uniform microwell platform resulted in homogeneity and easy control over the size of cell clusters. The proper cell cluster size improved the adequate supply of nutrients and cell interactions.

Furthermore, the effectiveness of synthetic mRNA (synRNA)-transfected hESCs for the generation of islet organoids has also been investigated. After the transfection of synRNA-PDX1 and synRNA-NKX6.1, hESCs were differentiated into PDX1^+^/NKX6.1^+^ cells with a mixture of PDX1^+^/NKX6.1^-^ and PDX1^-^/NKX6.1^+^ cells within three days [92]. The detection of the expression level of marker genes such as FOXA2 and SOX17 for definitive endoderm and HNF1B and HNF4A for primitive gut tube showed that this approach can only induce hESC differentiation into the primitive gut tube, which skipped the definitive endoderm step. Further differentiation was processed in a 3D culture system. The synRNA-transfected cells showed higher gene expression levels of insulin, glucagon, and somatostatin compared to the non-transfected cells, even though pancreatic polypeptide was not detected [92]. This differentiation procedure derives polyhormonal pancreatic endocrine cells with a mixture of mono-hormonal β-cells at day 13. Hence, the synRNA transfection strategy significantly accelerates the differentiation process to obtain islet-like organoids [92].

In another study, PI3K signaling has been examined for its efficiency in enhancing the differentiation of pancreatic islet cells. After the formation of pancreatic endocrine cells, cells were treated with LY294002, a type of nonselective PI3K inhibitor. It was found that the PI3K inhibitor significantly increased mRNA expression levels of insulin, somatostatin, pancreatic polypeptide, and glucagon [93]. Moreover, the expression of NKX6.1, MAFA, and GLUT2 was enhanced as well, suggesting that the derived cells were more mature after PI3K signaling was inhibited. The in vitro insulin secretion at a high glucose (25mM) level increased by four-fold compared to the cells treated with exendin-4. After transplantation in diabetic mice, the animals’ blood glucose remained stable and the cells survived even after 8 weeks [93].

### 7.2. ECM Signaling Enhances the Differentiation of Islet Organoids

ECM proteins provide cells with support and nutrition and help them interact with each other. They are important for controlling the proliferation, differentiation, and programmed death of stem cells [94]. The effect of ECM on in vitro hPSC differentiation into pancreatic islets has been extensively studied. The Brafman group confirmed that fibronectin- and vitronectin-coated substrates significantly promote the formation of definitive endoderm from hESCs compared to cells cultured and differentiated on Matrigel-coated substrates or other ECM proteins, such as collagen and laminin [95]. The combination of fibronectin and vitronectin showed enhanced performance for definitive endoderm formation, while also improving the efficiency of further differentiation into pancreatic endoderm [95].

The Wang group generated islet-like cell clusters from mouse embryonic stem cells in type I collagen scaffolds [96]. The porous structure of collagen gel provided a support for cells to grow into embryoid bodies first, followed by 25 days of differentiation. The scaffold differentiation platform resulted in approximately 50–60% of insulin^+^ cells, while less than 10% of cells cultured in the 2D environment were insulin^+^. Notably, the insulin secretion in cells differentiated inside scaffolds exhibited an approximately five-fold increase in a high glucose concentration compared to that in a low glucose concentration. In addition, the expressions of glucagon and somatostatin hormones were detected in cell clusters grown in scaffold culture environments [96]. The scaffold differentiation platform was applied to generate human islet-like organoids using hESCs [6]. In this study, type I collagen and Matrigel were used to form a porous, biocompatible scaffold for the differentiation of pancreatic islet organoids. Matrigel acts by adjusting and enhancing the mechanical properties of the scaffold since collagen itself lacks mechanical strength and is unstable due to cell contraction during stem cell differentiation. Of the three different concentrations of Matrigel examined, 10, 35, and 50% (*v*/*v*), the scaffold containing 35% Matrigel was found to have the best impact on differentiation efficiency. There was also a five-fold increase in insulin gene expression from cells cultured in scaffolds compared to that of 2D cultures. The 3D cultured cell clusters demonstrated glucose-responsive insulin secretion capacity. Remarkably, the expression of glucagon, somatostatin, and PP was also detected. The expression of other important gene markers like MAFA and GLUT2 was significantly increased as well [6].

Recently, Bi and his coworkers reported that the decellularized rat pancreatic ECM (dpECM) provides tissue-specific cues for hPSC pancreatic islet development [7]. The iPSC-derived islet organoids self-assembled into islet tissue architecture when they were exposed to the dpECM at the early stage of differentiation and switched to suspension culture during later stages of differentiation. These organoids consisted of four major types of endocrine cells, α-, β-, δ-, and PP-cells. In particular, these organoids were capable of secreting both insulin and glucagon in a glucose-responsive manner [7]. These experimental results unveiled the importance of natural and bioactive ECM as microenvironments for generating islet organoids from stem cells. The further characterization of protein contents of dpECM by mass spectrometric analysis and bioinformatics identified distinct signaling molecules in dpECM [8]. Among these molecules, type V collagen was found to be exclusively present in dpECM and not Matrigel. The exposure of iPSCs to type V collagen at the early stage of differentiation and suspension culture at the later stage led to the development of physiologically functional islets consisting of α-, β-, δ-, and PP-cells with a structure and cellularity similar to adult islets. Dual color immunostaining analysis revealed that the majority of β-cells were mono-hormonal expressing cells that expressed high levels of PDX1, insulin, NKX6.1, and MAFA at both gene and protein levels as compared to those cells generated in the absence of collagen V stimulation. Significantly, the organoids were capable of secreting insulin and glucagon in response to glucose level changes, suggesting a high degree of physiological function [8].

Other ECM proteins, such as laminin and fibronectin, have been reported to help form islet-like structures from the differentiation of mesenchymal stem cells [97]. It was speculated that these two ECM proteins can enhance the activation of Akt and extracellular-signal-regulated kinase (ERK) pathways. Cells cultured in ECM that consists of laminin and fibronectin showed the islet cells were able to release insulin in response to glucose concentration change [94]. Moreover, in a cell suspension culture system, the addition of fibronectin could also enhance the expression of proinsulin and insulin, and the cells secreted glucose-responsive insulin [94]. Therefore, a 3D culture model and signaling molecules are both indispensable for the generation of islet organoids from stem cells. It is speculated that 3D culture permits a higher degree of cell–cell interactions as compared to a 2D culture platform [7].

It should be pointed out that mesenchymal stem cells obtained from bone marrow or umbilical cord can be another renewable source to derive human endocrine cells [97,98]. Its low immunogenicity makes it available for clinical use [99]. A four-step protocol to differentiate human fetal bone mesenchymal stem cells into islet cells has been developed. After transplantation of the derived islet cell clusters into diabetic mice, the islet cells were able to control glucose levels in the mice for at least 9 weeks [99]. The generation of islet-like clusters from human umbilical cord mesenchymal stem cells has been explored. However, there was no indication that these islet-like clusters consisted of δ- and PP-cells [98]. Table 3 summarizes some methods to generate pancreatic endocrine cells from hPSCs. Most of them were able to produce glucose-responsive insulin-secreting endocrine cells. Only a few studies examined the glucose-stimulated glucagon secretion (GSGS) capacity of the iPSC-derived endocrine cells. In some animal studies, these islet cells have been proven to function for a relatively long time to balance blood glucose levels (Table 3).

## 8. Perspective

Our knowledge of the signaling molecules that promote pancreatic islet cell development from stem cell differentiation has been substantially enriched over the past two decades. Protocols ensuring the generation of insulin-secreting β-cells from hPSCs have been well established. Nevertheless, there are several limitations for pancreatic islet differentiation from iPSCs. Challenges in the generation of biologically functional islet organoids remain. First of all, the purity or the yield of functional islet organoids generated from hPSCs need to be improved. This is partially due to the lack of tools that can enrich islet organoids or their progenitors from all the cell aggregates generated during hPSC differentiation. In order to increase the purity of insulin-secreting β-cells from stem cells, fluorescence-activated cell sorting (FACS) technology has been employed to sort the derived β-cells and then aggregate them into about 100 µm cell clusters [100]. After further culturing, most cells were *C*-peptide-positive β-cells. The enriched β-like cell clusters showed glucose responsiveness similar to human islets, while cell clusters without the sorting step failed to show glucose level-regulated insulin secretion. However, a major drawback of FACS is that the process of sorting β-cells and their progenitors reduces cell survival rate. A second limitation is that the secretion levels of *C*-peptides or insulin are low and the enriched β-like clusters did not show a second-phase insulin response [100], implying that β-cell maturation is still a challenge. Thirdly, the efficiency of the generation of islet organoids remains low. There is no reliable method that can separate islet organoids from other cell aggregates at the end of stepwise iPSC progression into islets. In addition, the maturity of islet cells is problematic in most current differentiation protocols. Lastly, there exists an ultimate challenge, in that the overall hormone release capacity of the hPSC-derived islets is not comparable to that of adult islets. Furthermore, using iPSC lines to produce islet cells for personalized medicine is not straightforward. This is attributable to substantial variations among different iPCS lines, as they were derived from donors with varied ages and/or tissues, or different reprogramming protocols. These variations result in inconsistent differentiation efficiency when using one fixed differentiation protocol. Often, a protocol needs to be optimized for individual iPSC lines, which can be time- and labor-consuming. Hence, further studies may focus on developing not only an effective approach to boost hormone release capacity and glucose sensitivity, but also a robust and universal protocol for a variety of iPSC lines, making lab-generated islet organoids equivalent to native adult islets for clinical applications, disease modeling, and diabetes research.

## Figures and Tables

**Table 1:** Molecules that promote the maturation of β-cells.

Molecule	Function	Period	Cell Line	Timeline	Reference
T3	Increase MAFA expression	Stage 5	CyT49	Five stages (21+ days)	[23]
ALK5 inhibitor	Increase MAFA expression	Stage 5~7	H1	Seven stages (~40 days)	[2]
Exendin-4	Improve β-cell maturation	Stage 4	H9, H1	Four stages (22 days)	[4]
		Stage 5	H1, Epi-9, iPS1-10	Five stages (30 days)	[39]
RA	Increase PDX1, PAX4, and GLUT2 expression	Stage 3	MEL1/INS^GFP/W^	Five stages (20 days)	[40]
		Stage 3~5	HUES8, iPSC-1, iPSC-2	Five stages (~35 days)	[3]
Tankyrase inhibitor G007-LK	Inhibit Wnt signaling	Stage 7	ND41866	Seven stages (~40 days)	[41]

**Table 2:** Molecules that promote the maturation of α-cells.

Molecules	Function	Period	Cell Line	Timeline	Reference
LDN	Suppress NKX6.1 expression	Stage 3	HUES8, 1016	Five stages (48 days)	[63]
PDBu	Activator of PKC	Stage 5	HUES8, 1016	Five stages (48 days)	[63]
Noggin	Inhibit BMP signaling	Stage 2~3	H1	Six stages (26 days)	[68]

**Table 3:** Generation of pancreatic endocrine cells from pluripotent stem cells.

Reference	Stages (Days)	Strategy	Cell Line	GSIS (Fold Change)	GSGS (Fold Change)	δ Cells	PP Cells	In Vivo Study
Jiang et al., 2007 [5]	Four-stage (36)	2D	H1, H7, H9	3.3	N/A	N/A	N/A	N/A
Bi et al., 2020 [7]	Five-stage (28)	2D for 18 days; 3D suspension for 10 days	IMR90, H9	~2.8	2	Detected	Detected	N/A
Bi et al., 2020 [8]	Five-stage (28)	2D for 18 days; 3D suspension for 10 days	IMR90	~2.3	~4	Detected	Detected	N/A
Peterson et al., 2020 [63]	Five-stage (48)	Suspension culture	HUES8, 1016 cell	~2	~2	N/A	N/A	N/A
Massumi et al., 2016 [39]	Five-stage (30)	2D	H1, Epi-9, iPS1-10	~5.2	N/A	Detected	N/A	N/A
Wang et al., 2017 [6]	Four-stage (23)	3D collagen scaffold	H9	~3.5	N/A	Detected	Detected	N/A
Kim et al., 2016 [90]	Four-stage (17)	Suspension culture on the last day of differentiation	H1, CHA15	2.5	N/A	N/A	Not detected	Function in diabetic mice for 12 days
Hirano et al., 2017 [91]	Four-stage (31)	Suspension culture with closed-channel device	253G1	~4	N/A	Detected	N/A	N/A
Rezania et al., 2011 [68]	Seven-stage (26)	2D	H1	N/A	~2	Detected	N/A	Function in diabetic mice for over 90 days
Rezania et al., 2014 [2]	Seven-stage (30~40)	2D at Stage 1~4; 3D suspension at Stage 5~7	H1, homemade iPSCs	1.7	N/A	Detected	Not detected	Function in diabetic mice for 40 days
Pagliuca et al., 2014 [3]	Six-stage (30~37)	Suspension culture in spinner flasks	HUES8, homemade iPSCs	~3.0	N/A	N/A	N/A	Function in diabetic mice within 2 weeks
Mao et al., 2017 [93]	Five-stage (35)	2D	PKU1.1	N/A	N/A	Detected	N/A	Function in diabetic mice
Lin et al., 2010 [94]	Four-stage (10)	Suspension culture	Homemade hMSCs	~2	N/A	N/A	N/A	N/A
Chao et al., 2008 [98]	Four-stage (34)	2D	Homemade hMSCs	~5	N/A	N/A	N/A	Function in diabetic rats for over 21 weeks
